# Reduction of Suicidality and Depressive Symptoms with Esketamine: Focus on Gender Differences

**DOI:** 10.1192/j.eurpsy.2026.10589

**Published:** 2026-07-31

**Authors:** V. Martiadis, F. Raffone, S. Testa, C. Iaccarino, M. Russo, E. Pessina, A. Martini, M. Olivola, C. I. Cattaneo, D. De Berardis

**Affiliations:** 1 Department of Mental Health, Asl Napoli 1 Centro, Naples; 2 Department of Mental Health, Asl Cuneo 2, Bra; 3 UOC Psichiatria 1, ASST Fatebenefratelli-Sacco, Milan; 4 Department of Mental Health, Asl Biella, Naples; 5 Department of Mental Health, Asl Teramo, Teramo, Italy

## Abstract

**Introduction:**

Treatment-resistant depression (TRD) is a severe condition associated with high suicide risk and limited response to conventional antidepressants. Intranasal esketamine has shown rapid efficacy in reducing depressive symptoms and suicidal ideation.

**Objectives:**

This observational study assessed the real-world effectiveness of esketamine in reducing suicidal ideation, self-harm behaviors, and depressive symptoms in TRD patients, with a focus on potential gender differences in treatment response.

**Methods:**

We retrospectively analyzed the clinical records of 26 adults with TRD treated with intranasal esketamine at a specialized outpatient service between January 2023 and October 2024. Depressive symptoms were assessed using the Montgomery–Åsberg Depression Rating Scale (MADRS), while suicidal ideation and behavior were evaluated with the Columbia Suicide Severity Rating Scale (C-SSRS). Assessments were performed at baseline, week 2, and week 4. Gender-based response patterns were also analyzed.

**Results:**

Esketamine treatment resulted in significant reductions in suicidal ideation (C-SSRS: t = 12.36; p<.001) and depressive symptoms (MADRS: t = 10.87; p<.001) within four weeks. Non-suicidal self-injury decreased more rapidly in female patients (t = 2.71; p = 0.01), whereas male patients exhibited a slower but significant improvement in suicidal behaviors. A significant correlation was observed between reductions in depressive symptoms and suicidal ideation (τb = 0.419; p = 0.002), suggesting a close interaction between these domains.

**Conclusions:**

Esketamine demonstrated rapid effectiveness in reducing suicidality and depressive symptoms in TRD patients in a real-world setting. The observed gender-specific patterns in response highlight the need for personalized treatment approaches. Larger, prospective studies are warranted to confirm these findings and explore underlying mechanisms.

**Disclosure of Interest:**

None Declared

